# RNA polymerase pausing at a protein roadblock can enhance transcriptional interference by promoter occlusion

**DOI:** 10.1002/1873-3468.13365

**Published:** 2019-03-29

**Authors:** Nan Hao, Michael T. Crooks, Adam C. Palmer, Ian B. Dodd, Keith E. Shearwin

**Affiliations:** ^1^ Department of Molecular and Biomedical Science School of Biological Sciences The University of Adelaide Adelaide Australia; ^2^ CSIRO Synthetic Biology Future Science Platform Canberra Australia; ^3^ Department of Systems Biology Harvard Medical School Boston MA USA

**Keywords:** bacteriophage, mathematical modelling, promoter occlusion, RNAP pausing, transcriptional interference, transcriptional roadblocking

## Abstract

Convergent promoters exert transcriptional interference (TI) by several mechanisms including promoter occlusion, where elongating RNA polymerases (RNAPs) block access to a promoter. Here, we tested whether pausing of RNAPs by obstructive DNA‐bound proteins can enhance TI by promoter occlusion. Using the Lac repressor as a ‘roadblock’ to induce pausing over a target promoter, we found only a small increase in TI, with mathematical modelling suggesting that rapid termination of the stalled RNAP was limiting the occlusion effect. As predicted, the roadblock‐enhanced occlusion was significantly increased in the absence of the Mfd terminator protein. Thus, protein roadblocking of RNAP may cause pause‐enhanced occlusion throughout genomes, and the removal of stalled RNAP may be needed to minimize unwanted TI.

## Abbreviations

**MM**, minimal medium

**RBS**, ribosome‐binding sequence

**REO**, roadblock‐enhanced occlusion

**RNAP**, RNA polymerases

**TI**, transcriptional Interference

**Figure 1:**
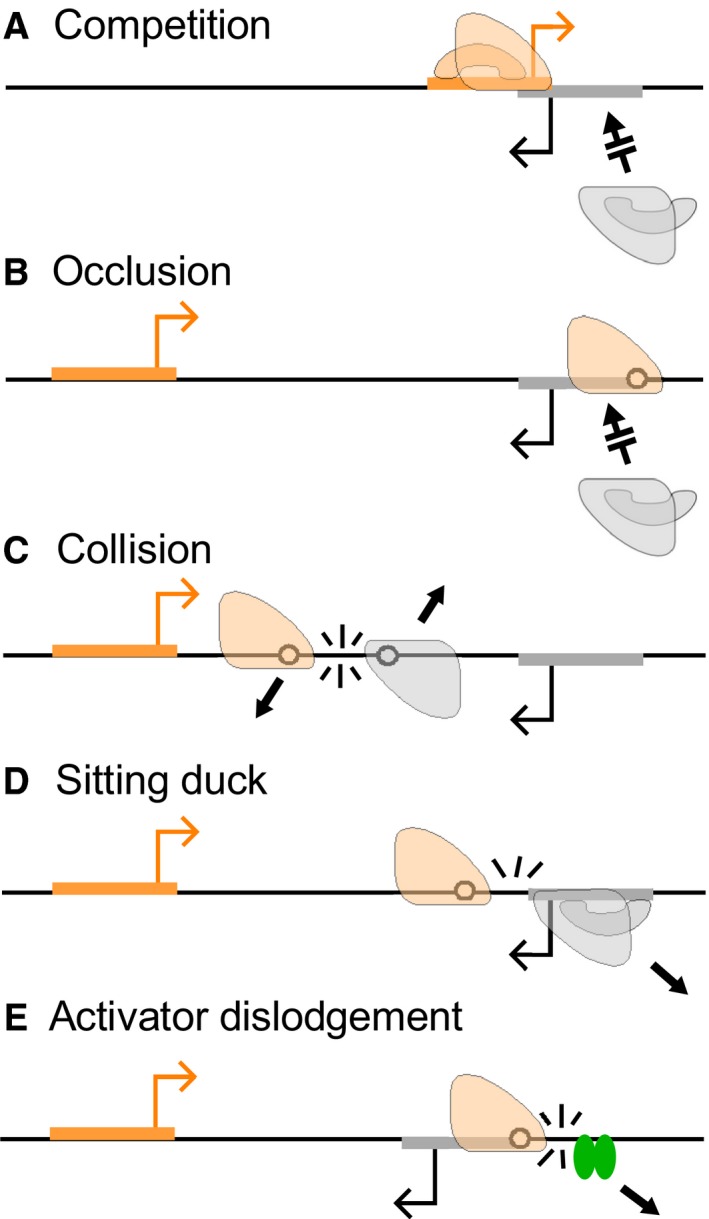
Five major mechanisms of transcriptional interference (TI) between convergent promoters.

Transcriptional interference (TI) is ‘the suppressive influence of one transcriptional process, directly and *in cis* on a second transcriptional process’, and is the result of RNA polymerase (RNAP) encountering promoters, DNA‐bound transcriptional factors or other RNAPs in the process of transcription 1. In prokaryotes, TI can arise *via* five major mechanisms (Fig. 1) 2, 3. For overlapping promoters, promoter competition (Fig. 1A) is the predominant TI mechanism. Promoter competition occurs as a result of the steric hindrance between two initiation complexes such that only one of the overlapping promoters can be bound by an RNAP at any given time. Such promoter arrangements are common, being found for ~ 14% of annotated *Escherichia coli* promoters 4. The remaining TI mechanisms – promoter occlusion, collisions and dislodgement of promoter‐bound RNAPs or activators – apply for non‐overlapping promoters, with all four mechanisms possible when the promoters are convergent (Fig. 1B–E) 2. Convergent promoter arrangements are also common, with over 1000 antisense transcripts identified as starting within *E. coli* coding regions 5. TI between convergent promoters increases with promoter strength (i.e. the flux of elongating RNAPs) but the different mechanisms are affected by RNAP flux and various other factors in different ways 6. Thus, the overall impact of TI and the primary mechanisms involved varies for each case.

Here, we focus on the promoter occlusion mechanism of TI, where elongating RNAPs positioned over a promoter prevent RNAPs in solution accessing the promoter (Fig. 1B). Because elongating RNAPs overlap a downstream promoter for only a short time, strong TI by occlusion requires either a very high flux of interfering RNAPs 6, or pausing of those RNAPs over the target promoter to increase occlusion time 7. Intrinsic RNAP pausing can be induced by specific sequences in the transcribed DNA or nascent RNA 8, 9, 10, 11 and occurs frequently in *E. coli* 12. We have shown that a strong pause site can significantly enhance occlusion 7. Since pausing can also be induced by DNA‐bound protein roadblocks 13, 14, we reasoned that a protein roadblock positioned such that the paused RNAP overlaps the target promoter would also enhance TI by promoter occlusion. Such roadblock‐induced pausing provides an alternative yet complementary avenue to study the pausing‐enhanced mechanism of TI, and may be a tool for manipulation of TI in regulatory circuits.

Collisional TI results from the termination of one or both elongating RNAPs when RNAPs from convergent promoters collide (Fig. 1C). Previous modelling of TI has suggested that collisions result in the removal of both RNAPs from the DNA 6, although imaging of convergent transcription *in vitro* has later suggested collisions might have diverse outcomes, with some RNAP stalling and remaining attached to the DNA and others being forced to backtrack 15. The magnitude of TI by collision depends on promoter separation; short promoter separations reduce the probability that an RNAP will be fired from the opposing promoter in the time it takes an RNAP to clear the region between the convergent promoters, and thus reduce collisional TI 6. Recent studies have indicated that RNAP loss after collisions can be asymmetric, favouring RNAPs whose transcripts are actively translated over RNAPs making RNA free of ribosomes 16, 17.

In sitting duck TI 18, an elongating RNAP from an opposing promoter removes RNAP at intermediate steps of initiation at the promoter (Fig. 1D), including stable closed complexes, open complexes and pre‐clearance initiation complexes 19. The magnitude of sitting duck interference felt by a promoter depends in part on its strength relative to that of the opposing promoter, and thus the overall amount of sitting duck TI experienced by a pair of convergent promoters is minimized when the promoters are of equal strength 6.

Dislodgement of activators by elongating RNAPs (Fig. 1E) has also been suggested as a form of TI 2, 7, as the consequent decrease in the occupancy of the activator's binding site should reduce activation of the target promoter. The magnitude of the loss of occupancy due to dislodgement is expected to be strongly affected by the DNA binding kinetics of the activator, with activators with slow binding and unbinding rates being more affected than activators with fast binding kinetics 2, 3.

**Figure 2:**
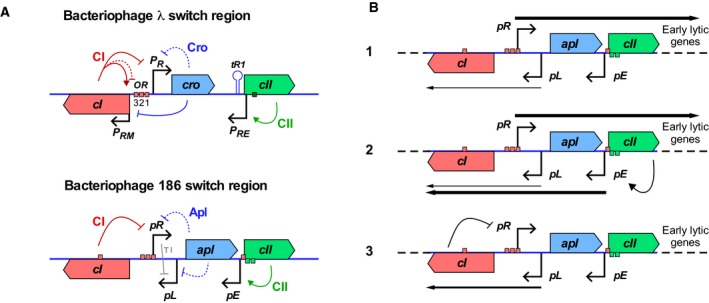
Regulation in the bacteriophage 186 *pR*‐*pE* region. (A) A comparison between phages 186 and λ lytic–lysogenic switch regions. The red and green boxes indicate the CI‐ and CII‐binding sites respectively. (B) Establishment of lysogeny in bacteriophage 186. (1) After infection, strong lytic transcription from *pR* inhibits *pL* by TI. (2) CII produced from *pR* activates *pE*, producing CI. (3) If sufficient CI is produced, *pR* is repressed, and the phage enters lysogeny. TI from *pR* at *pL* is alleviated, increasing *pL* activity and allowing *pL* to maintain CI production.

These convergent promoter mechanisms combine to produce a case of strong TI in bacteriophage λ, in which the lytic promoter *P*_*R*_ exerts ~ 6‐fold TI on the convergent lysogeny‐establishing *P*_*RE*_ promoter 7 (Fig. 2A). *P*_*RE*_ is activated by the λ CII protein and is necessary for production of sufficient CI immunity repressor to establish lysogeny after infection 20. Thus, inhibition of *P*_*RE*_ by *P*_*R*_ is likely to play an important role in the lysis–lysogeny decision of the phage. Pause‐enhanced occlusion is the major mechanism of TI in this case 7. *P*_*R*_ and fully activated *P*_*RE*_ are of similar strengths and are separated by just 320 bp, thus only moderate TI by the sitting duck and collision mechanisms would be expected. In addition, dislodgement of λ CII by RNAPs from *P*_*R*_ was found to not impact on CII activity 7. However, the weak *tR1* terminator induces pausing of *P*_*R*_ RNAPs over *P*_*RE*_, significantly enhancing TI by occlusion. It was proposed that RNAP pausing may be a widespread mechanism to enhance TI; however, no other examples of this mechanism have been observed.

The P2‐family *E. coli* bacteriophage 186, though essentially unrelated to λ, has a remarkably similar arrangement of the lytic‐ and lysogeny‐establishing promoters (Fig. 2A), with the *pR* lytic promoter and the 186 CII‐activated *pE* promoter convergent and separated by 340 bp. As fully activated *pE* is of similar strength to *pR*, and with a lack of known RNAP pausing at *pE*, weak TI would be expected. Indeed, less than twofold mutual TI between *pR* and *pE* was observed in initial experiments 21. However, TI at physiologically relevant lower induction of *pE* was not examined. Here, we varied the expression level of CII and thus *pE* activity, and found that substantial TI of ~ 4‐fold by *pR* on *pE* occurred when *pE* was only partially activated. The results could be explained by a model lacking any pause‐enhanced occlusion but in which the 186 CII protein was sensitive to dislodgement by RNAPs from *pR*.

We next tested roadblock‐enhanced occlusion in a synthetic construct with convergent promoters in which Lac repressor was used to stall RNAP such that it overlapped one of the promoters and thereby interfered with its transcription. We saw strong roadblock‐enhanced TI in an *E. coli mfd* mutant, in which termination of stalled RNAPs is defective 22, 23, including at protein roadblocks 13. However, the TI enhancement in *mfd* wild‐type cells was modest, with modelling indicating that roadblock‐enhanced occlusion is limited by rapid removal of RNAPs by Mfd unless the interfering promoter is strong.

## Materials and methods

### Strains and reporters

All *lacZ* reporter constructs used in the TI experiments (Fig. S1) were integrated into the λ *attB* site of *E. coli* strain NK7049 *(ΔlacIZYA)X74 galOP308 Str* ^*R*^ *Su* ^*−*^. The roadblock‐enhanced occlusion (REO) constructs (Fig. S1) were integrated into the λ *attB* site of *E. coli* MG1655 *rph+ ΔlacIZYA*, or a MG1655 derivative with an in‐frame deletion of the *mfd* gene 13. EC100D *mcrA Δ*(*mrr‐hsdRMS‐mcrBC*) φ80d*lacZΔ*M15 *Δlac*X74 *recA1endA1 araD139 Δ* (*ara,leu*)7697 *galU galK* λ^−^ *rpsL nupG pir* ^+^ (DHFR) (Epicentre, Madison, WI, USA) was used for propagation of *R6*γ*K ori* (*pir*‐dependent) plasmids.

DNA constructions used commercial DNA synthesis (Integrated DNA Technologies, Coralville, IA, USA), restriction enzyme‐based cloning and isothermal Gibson assembly.

The phage 186 *pR* and *pE lacZ* reporters for the TI experiments were based on the CRIM plasmid system 24. The *pR* to *pE* region was amplified from 186 phage DNA, and cloned into KpnI/SphI or KpnI/XbaI sites of *placatt1‐∆lacY‐lacZ* (Fig. S1). The *pR* and *pE* promoter mutants were generated by QuikChange mutagenesis. The activity of *pR* was suppressed by 186 CI expressed from pZC320 186 *cI* during the cloning process, as unrepressed multi‐copy *P*_*R*_ *‐lacZ* transcription led to cell lethality.

All REO reporters were derived from pIT3‐CL.lacZ* (Fig. S1). In this plasmid, the native ribosome‐binding sequence (RBS) of *lacZ* was weakened by mutagenesis, making it ~ 62 times weaker than that of the wild‐type *lacZ* RBS.

### DNA constructions

CII was expressed from pZS15_pET_RBS_*cII*, a low copy number (pSC101 origin) plasmid derived from pZS15 25. The expression of CII was controlled by LacI, expressed from the medium copy (p15A origin) pUHA‐1 plasmid (H. Bujard, Heidelberg University, Germany), and induced by isopropyl β‐d‐1‐thiogalactopyranoside (IPTG).

### LacZ assays

Microtiter plate‐based LacZ assays were carried out as previously described 13. Cultures were grown at 37 °C in microtiter plates until late log phase in either LB for TI experiments or M9 minimal medium (MM) for REO experiments. Twenty microlitres of culture was added to a combined lysis‐assay buffer, with each well of a microtiter plate containing: 30 μL culture medium (LB or MM), 150 μL of TZ8 (100 mm Tris/HCl, pH 8.0, 1 mm MgSO_4_, 10 mm KCl), 40 μL of ONPG (o‐nitrophenyl‐β‐d‐galactoside, 4 mg·mL^−1^ in TZ8), 1.9 μL of 2‐mercaptoethanol and 0.95 μL of polymyxin B (20 mg·mL^−1^). Assays were performed on cultures started from independent colonies and repeated on at least three different days. Error bars represent 95% confidence intervals.

### Stochastic simulations

Programs for stochastic simulation were written in FORTRAN and were executed on a MacBook Pro. In a typical run, 10^8^ time‐steps (~ 700 h) were simulated for each condition.

Rates are taken directly from previous simulations except where otherwise noted (Table S1). Promoter firing rates (*k*_F_) for the phage 186 *pR* and *pE* promoters used in the TI experiments were calibrated using the *P*_*Bla*_ promoter, for which *in vivo* firing rates have been estimated 26. Under rich medium growth conditions (LB), *P*_*Bla*_ fires approximately once every 55 s, 3.18 and 5.39 times slower than *pR* and CII‐activated *pE*, respectively, leading to *k*_F_ estimates of 0.0582 s^−1^ for *pR* and 0.0989 s^−1^ for activated *pE*.

After promoter firing, an RNAP that has just fired can sterically block the promoter from access by another RNAP, until the first RNAP has transcribed a distance equal to its length. This process is referred to as self‐occlusion. To account for self‐occlusion, a further correction was made (Eqn (1)):

(1) $${k_{F}}^{*}=\frac{1}{\frac{1}{k_{F}}-\frac{l}{\nu }},$$

where *k*_F_* is the intrinsic firing rate, *k*_F_ is the measured firing rate, *l* is the occlusion length of an elongating RNAP (30 bp) and ν is the elongation velocity (40 bp·s^−1^). After accounting for self‐occlusion, the final adjusted intrinsic strengths of *pR* and *pE* were calculated to be 0.0609 and 0.1072 s^−1^ respectively.

For REO experiments, cells were grown in M9 minimal medium instead of LB. It is known that promoter firing rates change with cellular growth rates 26. Thus, the promoter firing rates were recalibrated based on λ *P*_*L*_ firing rates measured under the same growth conditions 26. In minimal medium, both 186 *pR* and P2 *P*_*e*_ were about two times weaker than λ *P*_*L*_, estimated to fire approximately once every 10 s 26. After correcting for self‐occlusion, the final adjusted intrinsic firing rates for 186 *pR* and P2 *P*_*e*_ were calculated to be 0.0554 and 0.0527 s^−1^ respectively.

### Data transformation

The amount of LacZ expressed by very strong promoters with the native *lacZ* RBS can exceed the linear range of the LacZ assay. In a previous study 13, 11 promoter pairs of varying strengths, each expressing *lacZ* with either its native RBS or the weak RBS (lacZ*) were constructed and assayed, and a empirically derived rectangular hyperbola equation was used to transform the native RBS data to correct for the nonlinearity in the assay (Eqn (2)):

(2) $$\text{LacZ}\left(\text{transformed}\right)=\frac{32.32\times \text{LacZ}}{2120-\text{LacZ}}.$$

The LacZ data obtained for the phage 186 TI experiments were subjected to this transformation. The average LacZ produced by *pR(pE‐).lacZ* after background correction (against *pR‐(pE‐).lacZ*) over the IPTG concentration range was calculated to be ~ 23.44 transformed LacZ units, and the intrinsic firing rate for *pR* was 0.0609 s^−1^ or ~ 219.2 transcripts per hour in LB. One transformed LacZ unit was thus equivalent to 9.35 transcripts per hour. To align the experimental data with the stochastic simulations, the experimental LacZ data obtained for the 186 TI experiments were converted to the transcripts per hour units using this conversion factor.

For the REO experiments, a weak RBS version of *lacZ* reporter was used. We assumed that there was negligible nonlinearity in the observed LacZ activities assayed with the weak RBS and thus no data transformation was required to this data set. In minimal medium, the intrinsic firing rate for *pR* was 0.0554 s^−1^ or ~ 199.4 transcripts per hour, and the average LacZ produced by *pR(P*_*e*_ ¯).lacZ* was 72.33 units. Thus, one LacZ unit was equivalent to 2.76 transcripts per hour in this system.

### Statistical analysis

Measured lacZ values are presented as the mean ± the 95% confidence limits. The EC_50_ of *pE* induction curves with or without *pR* were calculated using the Hill function in graphpad prism (Graphpad Software, San Diego, CA, USA).

## Results

### Design and construction of a bacteriophage 186 *pR*‐*pE* reporter system

The bacteriophage 186 lysis–lysogeny decision is regulated by three promoters: *pR*,* pE* and *pL*, and two regulatory proteins: CI and CII (Fig. 2A). Unlike λ, where the *P*_*R*_ lytic promoter and the *P*_*RM*_ lysogenic promoter are arranged back‐to‐back, the 186 lytic promoter *pR* and the lysogenic promoter *pL* are convergent. This arrangement results in 5.6 fold TI at *pL* primarily by the sitting duck mechanism 6, 18. During lysogeny, CI repression of *pR* indirectly activates *pL* by removing this TI, allowing for expression of lysogenic genes including *cI* 27, 28 (Fig. 2B, panel 3). However, upon phage infection of a cell that contains no CI protein, *pL* transcription is low due to TI and thus establishment of lysogeny requires an alternative source of CI (Fig. 2B, panel 1).

Efficient establishment of lysogeny is dependent on the 186 CII protein 29, which activates transcription of *cI* from the *pE* promoter 30, 31, 32 (Fig. 2B, panel 2). CII contains a helix‐turn‐helix motif and is highly unstable *in vivo*, with an estimated half‐life of 2.6 min 32. CII binds an inverted repeat spaced two turns of the DNA helix apart, located at the −38 and −58 positions of *pE,* and contacts both the α and σ subunits of RNAP 32. Basal *pE* is of negligible strength, but is strong when induced by CII 30.

A previous study examined TI between *pR* and *pE* under conditions where *pE* was fully activated with a high level of CII expression. Weak TI was seen, with *pR* reducing the activity of *pE* 2.1‐fold and *pE* reducing the activity of *pR* 1.5‐fold 21, suggesting a lack of substantial pause‐enhanced occlusion in the 186 case. However, given the strong interference exerted by λ *P*_*R*_ on *P*_*RE*_, we wished to test whether TI by 186 *pR* on *pE* might be stronger at lower *pE* induction levels.

In order to study the effect of *pE* induction level on the TI between *pR* and *pE*, a set of chromosomally integrated *lacZ* reporters was constructed (Fig. S1). The region of the 186 genome (NC_001317.1) used for these reporters spans from base 22 980, which is −81 from *pR*, to base 23 533, which is −132 from *pE* and ~ 70 bp from the end of the CII‐binding site (Fig. S2). This region of the 186 genome also contains the *pL* promoter and the full coding sequence of *apl*, both of which were mutationally inactivated by (a) altering the −10 site of *pL* 18 and (b) swapping residues E28 and R29 of Apl that lie within the recognition helix of Apl's helix‐turn‐helix motif. Together, these alternations are designed to retain the distance between *pR* and *pE* and to minimize changes to the native 186 DNA sequence. While the *pL* ^*−*^ mutation was designed to avoid affecting *apl* translation, we found that Apl expression was significantly reduced, consistent with disruption of the *apl* ribosome‐binding site (Fig. S3).

**Figure 3:**
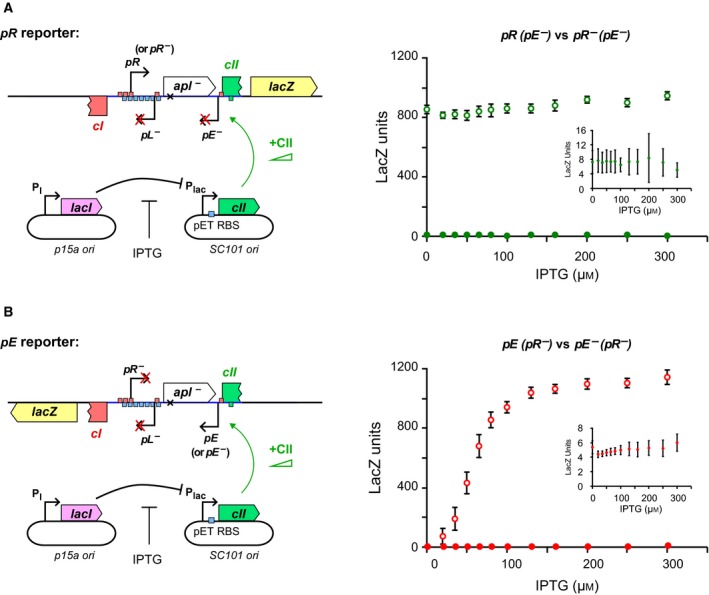
The effect of varying CII levels on *pR* and *pE* activity. (A) Left, a schematic representation of the chromosomally integrated *pR*(*pE‐*).*lacZ* and *pR‐*(*pE‐*).*lacZ* reporter constructs. CII was expressed from low copy number pZS15_pET_RBS_*cII* and was controlled by IPTG. Right, the *pR* activities were assayed across 12 IPTG concentrations from 0 to 300 μm. *pR* was constitutive and approximately constant, averaged across all IPTG concentrations at 862 ± 9 LacZ units (*n* = 238). Error bars represent 95% confidence intervals. Right insert, a zoomed in view of *pR‐*(*pE‐*).*lacZ* at different IPTG concentrations, showing that the mutation of *pR* almost completely abolished *P*_*R*_ activity. Open circles: *pR*(*pE‐*).*lacZ* data; filled circles: *pR‐*(*pE‐*).*lacZ* data. (B) Left, a schematic representation of the chromosomally integrated *pE*(*pR‐*).*lacZ* and *pE‐*(*pR‐*).*lacZ* reporter constructs. Right, the *P*_*E*_ activities across the IPTG range. *pE* was activated by the induction of CII expression with IPTG, from a basal activity of 5.1 ± 0.4 LacZ units to 1140 ± 50 lacZ units (*n* = 16). Right insert, a zoomed in view of *pE‐*(*pR‐*).*lacZ*. The *P*_*E*_ promoter mutation is a single base pair change at the −10 site of *pE*, which almost completely knocks out CII‐dependent *pE* activation but does not alter CII binding at *pE* 31. Note that the binding half sites of CII are centred at −38 and −58 of *pE* (Fig. S2). Open circles: *pE*(*pR‐*).*lacZ* data; filled circles: *pE‐*(*pR‐*).*lacZ* data.

The CII protein was expressed *in trans* from a plasmid‐based LacI‐repressed system with IPTG induction (Fig. 3). The pZS15_pET_RBS_*cII* plasmid (Materials and methods) is capable of producing a gradient of CII expression, up to the levels necessary to maximally activate *pE*, while minimizing leaky CII expression and changes in cell growth due to a high level of CII.

The *pR* and *pE* activities were first measured in the absence of the convergent promoter by constructing promoter null mutants (Fig. 3). The *pR‐* mutant was made by altering the −10 and −35 sites of *pR* 18 (Fig. S2), while the *pE‐* mutation is a single base substitution in the −10 site of *pE* (Fig. S2), which does not alter CII binding 31. In the absence of *pE*, transcription from *pR* was strong, constitutive, and not affected by the concentration of CII (Fig. 3A), indicating that DNA‐bound CII is not a transcriptional roadblock for elongating RNAPs from *pR*. In the absence of *pR*,* pE* had almost negligible basal activity but was strongly induced by IPTG‐regulated CII expression, reaching even greater activity than *pR* (Fig. 3B).

### Assaying TI between *pR* and *pE*

**Figure 4:**
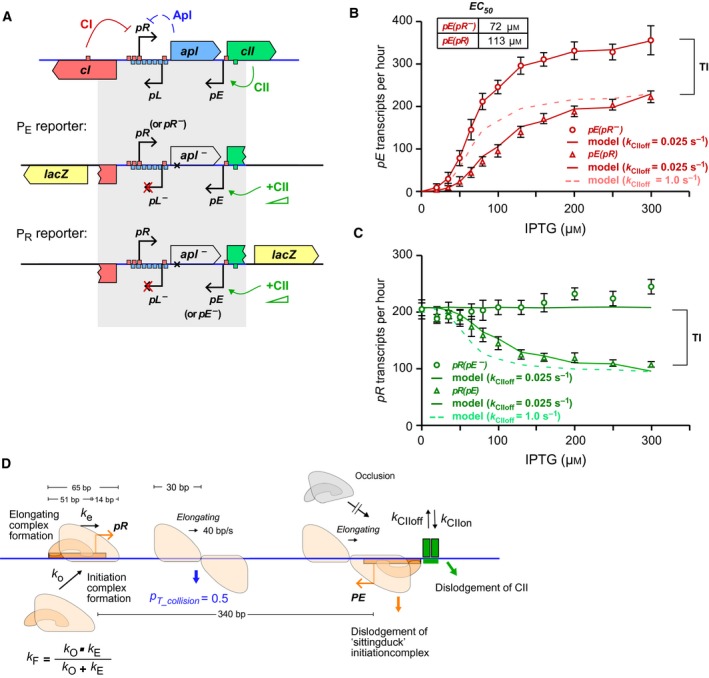
The convergent *pR* and *pE* promoters of bacteriophage 186 only weakly interfered with each other's transcription, even at high CII concentrations. (A) A schematic representation of the chromosomally integrated reporter constructs used to measure the activities of *pR* and *pE* with and without the convergent transcription from the other. (B) Data (symbols) and simulation (solid curve) for the activity of the *pE* in the *pE*(*pR‐*).*lacZ* and *pE*(*pR*).*lacZ* reporters with increasing concentration of CII. Data are mean ± 95% confidence intervals (*n* = 14). The dotted curve shows the simulation of *pE*(*pR*) with faster binding kinetics for CII (*k*_CIIoff_ = 1 s^−1^). (C) Data and simulation (solid curve) for the activity of *pR* in the *pR*(*pE‐*).*lacZ* and *pR*(*pE*).*lacZ* reporters. The dotted curve showed the simulation of *pR*(*pE*) with faster CII binding kinetics, as in (B). For (B, C), the activity was expressed in units of transcripts per hour to allow direct comparison with the simulations. (D) Schematic of the stochastic model and associated parameters used to simulate the experimental data.

Next we tested the activities of *pR* and *pE* when each promoter faced convergent transcription from the other (Fig. 4A). When *pR* was active, the activity of *pE* was reduced at all CII concentrations. The maximal *pE* activity was 1.63 (1.46–1.82) (95% confidence interval) fold less than that obtained when *pR* was mutationally inactivated (Fig. 4B). However, TI by *pR* on *pE* was stronger at lower CII induction levels, with 3.98 (2.96–5.50)‐fold TI at 50 μm IPTG (Fig. 4B). This effect substantially increased the IPTG concentration required to reach half‐maximal *pE* activation (the EC_50_), (from 72 μm without interference to 113 μm with interference), reflecting a requirement for higher CII expression levels (Fig. 4B). This change in EC_50_ is consistent with dislodgement of CII bound at *pE* by RNAP from *pR*. Furthermore, induction of *pE* by CII also caused a dose dependent reduction of *pR*, reflecting increased TI from a stronger *pE* (Fig. 4C). When *pE* was maximally activated, TI reduced *pR* about 2.27 (2.12–2.43)‐fold. The somewhat weaker 1.5‐fold inhibition of *pR* by *pE* seen previously 21 is likely to be due to non‐maximal *pE* activation.

### Modelling TI between *pR* and *pE*

To test if the observed TI between *pR* and *pE* can be explained by the known mechanisms of TI (Fig. 1), a mathematical model was developed based on our previous TI model, but altered to describe the convergent *pR* and *pE* promoters of phage 186 (Fig. 4D). A section of DNA from the −51 position of *pR* to the −51 position of *pE* was simulated. The model used the method of discrete fixed time‐steps to stochastically simulate the kinetics of RNAP and CII binding, as well as sitting duck, collision and occlusion TI on this DNA. In this simulation, each time‐step is set to 1/40 s, equivalent to the time taken for an elongating RNAP to advance one base pair 6. All possible events are assigned a specific rate *k* (Table S1). Whether or not a given event occurs during the next time‐step is decided by generating a random number between 0 and 1; if this number is less than 1 − e^−*k/*40^, then that event occurs. The simulation is updated to reflect this change, and the simulation proceeds to the next time‐step. Wherever possible, rates were taken directly from *in vivo* measurements from the literature. For the rates where no direct measurements are available, previously fitted rates were used, except where otherwise noted.

The key events in the simulation are as follows:

1. *Promoter firing*: Promoter firing is simulated as a two‐step process, consisting of loading of RNAP holoenzyme to form an open complex and firing of the open complex to form an elongating complex. Binding of an open complex to the promoter (defined as positions −51 to +14) is only possible when the promoter is not already overlapped by other RNAPs. A new open complex is loaded at *pR* with rate *k*_O_ = 0.061 s^−1^ or at a CII‐bound *pE* with rate *k*_O_ **= 0.214 s^−1^. No open complex can be formed at *pE* if the CII‐binding site is unoccupied by CII. An open complex is converted to an elongating complex with rates *k*_E_, which are 61 and 0.214 s^−1^ respectively for *pR* and *pE*. Unlike *k*_O_, the *k*_E_ for *pE* is CII independent, meaning that the *k*_E_ for *pE* will not change even if CII dissociates after an open complex has formed at *pE*. After promoter firing, the open complex is converted to an elongating complex, and its footprint reduced from 65 bp for an open complex to 30 bp 33.
2. *Binding and unbinding of CII at pE:* Binding of CII at *pE* only occurs if the CII site is not already occupied by CII, and is not overlapped by RNAPs from *pR*. The binding rate of CII (*k*_CIIon_) depends on its concentration (Table S1), which can be extracted from the un‐interfered *pE* induction curve (Fig. 3B) by assuming that activity is proportional to CII occupation and that the CII site is fully occupied at the maximal *pE* activity. Since occupation equals the association rate *k*_CIIon_ over the sum of *k*_CIIon_ and the dissociation rate *k*_CIIoff_, these data alone give a fix on the ratio of *k*_CIIon_ and *k*_CIIoff_ at each CII concentration, but does not provide any fix on the exact values of *k*_CIIon_ or *k*_CIIoff_. In the presence of *pR*, a DNA‐bound CII at *pE* either dissociates spontaneously with rate *k*_CIIoff_ or is removed by RNAPs initiated from *pR*. Dislodgement of CII by RNAPs was assumed to be instantaneous as CII is not a transcriptional roadblock to RNAP from *pR* (Fig. 3A). Thus, the *pR* interfered *pE* induction curve (Fig. 4B) puts a further constraint on *k*_CIIoff_. If *k*_CIIoff_ is fast, then dislodgement of CII by RNAPs will not have a large influence on the CII occupation; alternatively, if *k*_CIIoff_ is slow, then dislodgement by RNAP will have a pronounced effect on the CII occupation.
3. *Movement and termination of RNAPs:* RNAP elongation is treated as occurring at a constant rate at 40 bp·s^−1^ 6, consistent with measurements of the average speed of RNAP *in vivo* 34. RNAP velocity heterogeneity 35 was not simulated. In the model, collision of two convergent elongating RNAPs results in one (at random) being instantaneously removed, while the other remains (collision TI). Our results with synthetic TI constructs 16 indicated that RNAP loss after collisions is highly asymmetric between RNAPs whose RNAs are strongly translated and RNAPs whose RNAs are untranslated, with a 7% : 93% translated:untranslated removal ratio. However, while the *pR* transcripts would normally be translated over the *apl* coding sequence and the *pE* transcripts are untranslated, we did not include asymmetric removal of RNAPs in the model because the *pL* ^*−*^ mutation used in the constructs disrupts the *apl* ribosome‐binding site (Fig. S3). If the collision occurs between an elongating RNAP and an open complex, then the open complex is removed (sitting duck TI). Once the back of an elongating RNAP passes the end of the DNA, then that RNAP is eliminated from the DNA and a new transcript is counted.

The simulations use CII occupation as the independent variable versus transcripts per hour as the dependent variable, whereas the experimental measurements use IPTG concentration and lacZ activity respectively. To align the curves produced by simulation with the experimental values, the experimental LacZ activities were converted to transcripts per hour as described in the Materials and methods.

The model provided a reasonable fit to the data and reproduced the observed increase in EC_50_ as long as the *k*_CIIoff_ of CII was set low at 0.025 s^−1^, so that CII dislodgement becomes a significant effect (Fig. 4B,C). Thus, modelling suggests that a DNA‐bound CII takes on average ~ 40 s to spontaneously leave the DNA or a half‐life of ∼28 s (= ln2/*k*_CIIoff_). If the dissociation rate of CII is increased 40‐fold to 1 s^−1^ (with a compensating increase in *k*_CIIon_ to maintain occupation), inhibition of *pE* by *pR* is underestimated, especially at low CII concentrations (Fig. 4B, dotted line). In this case, the spontaneous rate of CII dissociation is high enough that the additional dissociation due to dislodgement by RNAPs from *pR* is minor and has little effect on CII occupation. The faster CII binding kinetics also produces too much inhibition of *pR* by *pE* (Fig. 4C, dotted line) as result of the higher *pE* activity.

**Figure 5:**
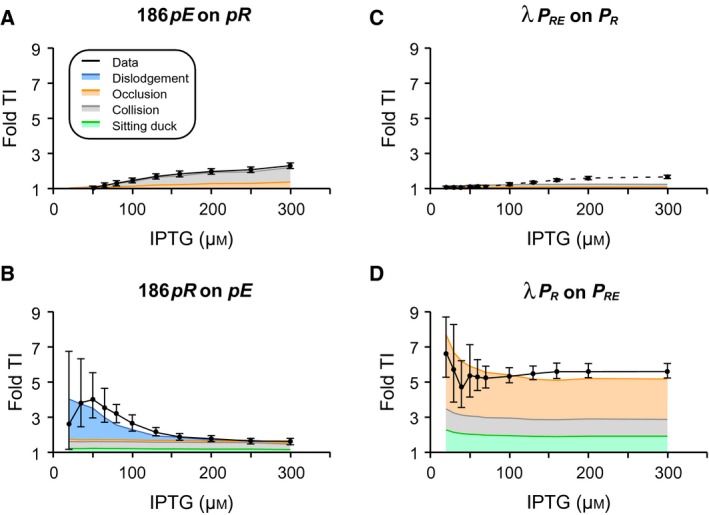
Bacteriophages 186 and lambda have evolved different mechanisms to induce TI between the convergent promoter pairs *pR*/*pE* and *P*_*R*_/*P*_*RE*_. (A) TI of 186 *pE* on *pR* is weak and predominantly *via* RNAP collision (B) TI of 186 *pR* on *pE* is stronger and peaks at low CII concentration as a result of CII dislodgement by transcription from *pR* (C) λ *P*_*R*_ experiences very little TI from *P*_*RE*_. (D) In contrast, λ *P*_*RE*_ experiences strong TI from *P*_*R*_, and is due to RNAP pausing at the *tR1* terminator overlapping *P*_*R*_. The black solid curves show the fold TI calculated from experimental data, error bars represent 95% confidence intervals (*n* ≥ 6). The λ TI data were calculated from Palmer *et al*. 7. The different colour shadings indicate the contribution of the different TI mechanisms.

Figure 5 shows how the measured TI between 186 *pR* and *pE* changes with induction of CII expression. The maximal observed TI by 186 *pE* on *pR* was ~ 2.3‐fold at the highest [IPTG] (Fig. 5A). The modelling allowed us to extract the relative proportions of the different TI mechanisms as CII concentration increased. This analysis (Fig. 5A) indicates that RNAP collisions were responsible for the majority (~ 75%) of the TI at all IPTG concentrations, and that promoter occlusion contributed to most of the remainder. The absence of sitting duck interference results from the very low aspect ratio (that is the rate of promoter binding over the rate of firing) assigned to *pR* (Table S1), which was necessary to fit the data.

The measured TI of 186 *pR* on *pE* peaked at ~ 4‐fold at 20 μm IPTG when *pE* activity was weak, and then gradually reduced to ~ 1.6‐fold when *pE* started to gain strength (Fig. 5B). Modelling indicates that at low IPTG concentrations the TI was largely due to CII dislodgement by elongating RNAPs from *pR*, contributing up to 75% of observed TI at 20 μm IPTG. As expected, TI due to CII dislodgement became diminished at higher CII concentration, and the TI became predominately due to RNAP collision followed by sitting duck and promoter occlusion. Note that although the percentage contribution of each TI mechanism changed at different IPTG concentrations, the absolute TI due to RNAP collision, sitting duck and promoter occlusion mechanisms remained constant across the IPTG range.

For comparison, Fig. 5C,D shows how TI between λ *P*_*R*_ and *P*_*RE*_ changes with induction of λ CII 7. The separation of λ *P*_*R*_ and *P*_*RE*_ is 320 bp, similar to the 340 bp between 186 *pR* and *pE*. The establishment promoters are also of similar strength, with firing rates of once every 8.13 s for λ *P*_*RE*_ compared to once per 9.3 s for 186 *pE*, while λ *P*_*R*_ (once per 5.8 s) is ~ 2‐fold stronger than 186 *pR* at once per 14.5 s. However, a key difference is that RNAPs from *P*_*R*_ pause for substantial periods at three sites within *tR1* 7. Interference by *P*_*RE*_ on *P*_*R*_ was at most 1.6‐fold (Fig. 5C), which is slightly lower than the 2.3‐fold for 186 *pE* on *pR* (Fig. 5A), primarily because of the higher strength of λ *P*_*R*_. However, in λ, interference at *P*_*RE*_ was very strong, peaking at ~ 6.7‐fold at low λ CII concentration and staying at ~ 5.5‐fold TI even at high λ CII concentration, with pause‐enhanced occlusion the major TI mechanism 7 (Fig. 5D).

Thus, despite the similar promoter arrangements in 186 and λ, the two phages display different mechanisms and magnitudes of TI. While we have no direct measurements of transcriptional pausing in 186, our data and modelling indicate a lack of significant pause‐enhanced occlusion at 186 *pE*. The addition of substantial pause‐enhanced occlusion would increase TI of 186 *pR* on *pE* across all IPTG concentrations and would substantially worsen the match to the observed TI at high [IPTG]. Increased occlusion would also tend to dampen the observed EC_50_ shift, which is instead explained by 186 CII's sensitivity to dislodgement.

### A roadblock‐enhanced occlusion circuit

While pause‐enhanced occlusion at λ *P*_*RE*_ produces a substantial regulatory effect, the apparent lack of pausing‐enhanced occlusion in the 186 *pR*–*pE* system raises the question of whether this mechanism applies in other cases. There are three clustered pause sites at λ *tR1* that produce an unusually long dwell time for RNAPs over *P*_*RE*_. Since the occlusion effect is strongly dependent on the RNAP pause time, it is possible that λ is a special case, and more typical pause sites may be unable to cause TI enhancement.

**Figure 6:**
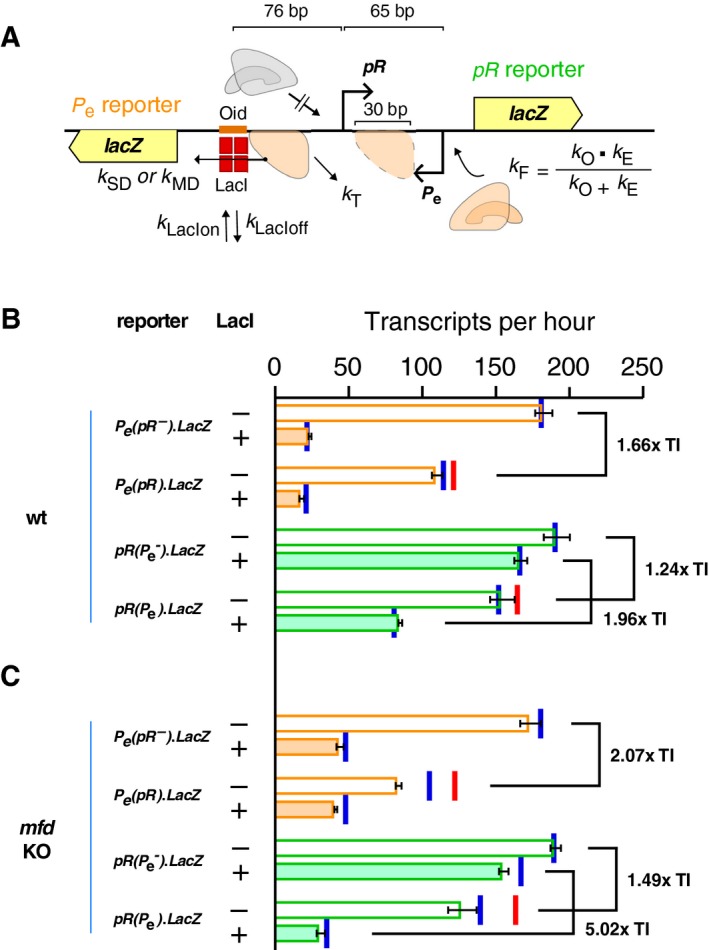
The roadblock‐enhanced occlusion (REO) circuit is a synthetic circuit with an engineered RNAP pause site at *pR* for the augmented induction of asymmetrical TI between convergent promoters *pR* and *P*_*e*_. (A) Schematic arrangement and parameters used for simulation of the REO circuit. The DNA region simulated in the modelling extends from the −85 position of 186 *pR* to the −51 position of P2*Pe*. (B) Data (horizontal bars) and simulations (red and blue vertical lines) for the TI between *pR* and *P*_*e*_ promoters with (filled bars) or without (empty bars) a LacI roadblock in the *mfd* wild‐type strain (C) as for (B), but reporters integrated into a *mfd* knockout strain. To align the experimental data with the simulations, data were converted to transcripts per hour units (Materials and methods), error bars represent 95% confidence intervals (*n* = 9). Red bars represent the simulated TI when collision‐induced termination was considered instantaneous, while blue bars represent the TI when the rate of collision‐induced termination was set equal to the rate of termination at the lacI roadblock.

To test whether other pauses can enhance TI, we used a protein roadblock, specifically the Lac repressor (LacI), to pause RNAP at one of a pair of convergent promoters (Fig. 6A and Fig. S4). LacI is the best studied of a handful of DNA‐binding proteins that are known to block the progress of elongating RNAP *in vitro* and *in vivo* 13, 14, 34. We expected that LacI bound to its strong *Oid* operator located just upstream of one of the promoters would cause stalling of RNAPs from the second promoter such that they block access to the first promoter (Figs. 6A and Fig. S4). A potential advantage of using a protein‐induced pause is that controlling the availability or activity of the roadblocking protein should allow ready modulation of the occlusion effect.

Our roadblock‐enhanced occlusion (REO) circuit system utilized the lytic promoters *pR* and *P*_*e*_ of phages 186 and P2 as the convergent promoter pair. The use of two similar strength promoters (186 *pR* 0.0554 s^−1^ and P2 *P*_*e*_ 0.0527 s^−1^) was designed to minimize sitting duck TI. The two promoters were also placed very close to each other (65 bp between the +1 of each promoter) to minimize TI by RNAP collision 6. The *lacOid* operator was centred at the −76 position of *pR*, a location where LacI binding had minor effects on *pR* activity. Four chromosomally integrated *lacZ* reporters were constructed (Fig. S1), two of which were used to report the activities of *pR*, either in the presence or absence of a convergent *P*_*e*_, and the other two reported the activities of *P*_*e*_ with or without a convergent *pR* (Fig. 6B). The Lac repressor was expressed from a medium copy number plasmid (pUHA‐1), under the control of its native promoter *P*_*I*_ 25. A Δ*lacI* version of the same plasmid was used as a no roadblock control 13.

In the absence of LacI, transcription from *pR* led to ~ 1.7‐fold TI on *P*_*e*_. Conversely, transcription from *P*_*e*_ led to ~ 1.2‐fold TI on *P*_*R*_ (Fig. 6B). This small difference in TI between *P*_*R*_ and *P*_*e*_ was probably due to the intrinsic kinetic parameters of the two promoters (Table S1). When LacI was present, the expression of *lacZ* from *P*_*e*_ was reduced to only ~ 13% of its normal level due to transcriptional roadblocking by LacI (Fig. 6B). This drop in transcription occurs because RNAPs stalled at the LacI roadblock are subject to termination 13. The effect of LacI on transcription from the *pR* promoter in the absence of convergent transcription from *P*_*e*_ was mild, reducing *pR* by ~ 10% (Fig. 6). However, in the presence of LacI, *P*_*e*_ produced a ~ 2.0‐fold inhibition of *pR* (Fig. 6B). We attribute this *P*_*e*_‐dependent inhibition of *pR* by LacI to enhanced occlusion of *pR* due to RNAP pausing at the LacI roadblock.

The pausing time of RNAPs at a protein roadblock is affected by the transcription‐coupled repair protein Mfd. The Mfd translocase moves unidirectionally along DNA and upon encountering a stalled or backtracked RNAP either stimulates reinitiation of elongation by pushing RNAP forward, or, if a strong obstacle prevents this, stimulates termination of the RNAP 22, 23. Both outcomes should result in a reduction of the pause time. At strong protein roadblocks, such as LacI, increased termination due to Mfd leads to reduced transcription past the roadblock 13, 23. Thus, to test the idea that the *P*_*e*_‐dependent inhibition of *pR* by LacI is due to enhanced occlusion, we assayed the reporters in an Δ*mfd* background (Fig. 6C). As expected, removal of Mfd increased *P*_*e*_ readthrough of the LacI roadblock, from 13% to ~ 25%. Importantly, the LacI enhancement in TI by *P*_*e*_ on *pR* increased dramatically from ~ 2‐fold to ~ 5‐fold in the *mfd* mutant (Fig. 6C), supporting the roadblock‐enhanced occlusion mechanism. Interestingly, the mutual TI between *P*_*R*_ and *P*_*e*_ in the absence of the LacI roadblock also increased slightly from ~ 1.2‐ to ~ 1.5‐fold and from ~ 1.7‐ to ~ 2.1‐fold, respectively, in the Δ*mfd* strains (Fig. 6C).

### Modelling the roadblock‐enhanced occlusion circuit

To test the roadblock‐enhanced occlusion mechanism further, we simulated the REO circuit, using a TI model modified to incorporate our previous transcriptional roadblocking model 13. In the model (Fig. 6A), an elongating RNAP becomes paused when it encounters a bound LacI roadblock. A paused RNAP stays paused unless it either spontaneously dissociates from DNA with rate *k*_T_, or dislodges the bound roadblock with rate *k*_SD_. If there is more than one RNAP paused behind a protein roadblock, then an increased dislodgement rate *k*_MD_ is applied to account for RNAP cooperation 34. The same treatment was applied to both the wild‐type and the *Δmfd* strain, but with different values for *k*_T_, *k*_SD_, and *k*_MD_ 13. All rates were as previously estimated except the *k*_T_ in the *Δmfd* strain, which was increased ~ 4.4‐fold from 0.0045 to 0.02 s^−1^, a necessary adjustment required to fit the somewhat stronger than expected roadblocking of LacI on *P*_*e*_(*pR‐*)lacZ* (Fig. 6C).

Overall, the model was able to reproduce the wild‐type data reasonably well (red bars, Fig. 6B,C). However, we found that the model underestimated TI for both *pR* and *P*_*e*_ in the Δ*mfd* strains. This may be a result of how RNAP termination was simulated after a head‐on collision between RNAPs. As previously stated, the model treats the dissociation of collided RNAPs as an instantaneous process, a reasonable simplification for the wild‐type cells. However, it is possible that the removal of RNAPs after collision is delayed in the *Δmfd* cells, given the role Mfd plays in the resolution of RNAPs stalled at other obstacles 23. Indeed, when the rate of collision‐mediated termination was delayed by setting it equal to the *k*_T_ used for RNAP termination at the LacI roadblock (0.02 s^−1^ for *Δmfd* cells and 0.66 s^−1^ for *mfd* ^*+*^ cells), the model was able to provide a better fit, particularly to the *Δmfd* data (blue bars, Fig. 6B,C). Because *pR* and *P*_*e*_ are very close, this delay in termination causes promoter ‘clogging’, where queues of RNAPs extend back from a collision event to cover the promoters and prevent loading of new RNAPs 13. This additional inhibition of transcription due to collisions increases TI. The effect is minor in wild‐type cells because rapid termination by Mfd keeps RNAP queues to a minimum.

**Figure 7:**
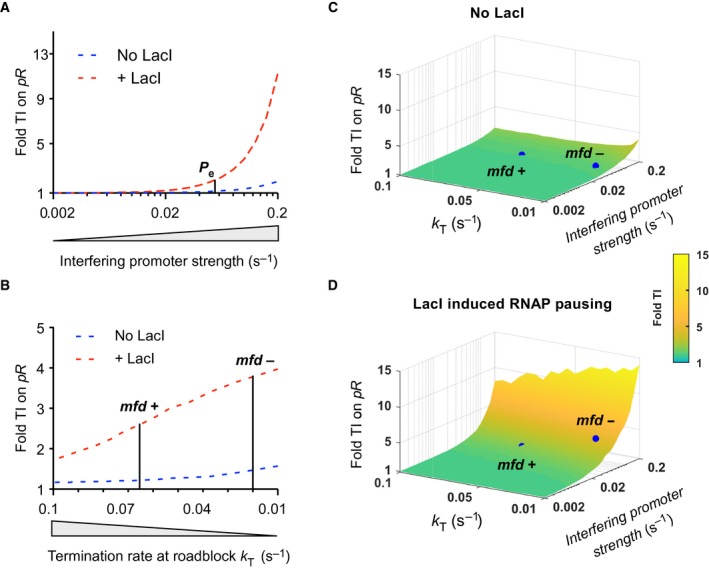
The strength of occlusion‐enhanced TI is dependent on both the strength of the promoter that controls RNAP flux at the roadblock site, and the rate of RNAP termination at the roadblock site. (A) Fold TI on *pR* increased with increasing *P*_*e*_ strengths (i.e. increased firing of RNAP towards the roadblock). *pR* strength (0.055 s^−1^) and RNAP termination rate (0.066 s^−1^) were held constant. (B) Fold TI on *pR* increased as the RNAP termination rate at the roadblock site was lowered (i.e. reduced loss of RNAP at the roadblock). *pR* and *P*_*e*_ strengths (0.055 and 0.053 s^−1^ respectively) were held fixed. (C) Roadblock‐enhanced occlusion on *pR* is maximized with a stronger *P*_*e*_ and a reduced RNAP termination rate at the roadblock site. Blue dots show the experimentally determined TI on *pR* in the absence (C) or the presence (D) of the roadblock protein, LacI.

The modelling allowed us to assess the impact of system properties on the strength of roadblock‐enhanced occlusion in the REO circuit. Given a strong roadblock, the main factors are the strength of the promoter supplying RNAPs to the roadblock, and the rate of termination of RNAPs stalled at the roadblock. It is the balance between this gain and loss of paused RNAPs that determines the fractional occupancy of the occluding site. Figure 7A shows how the TI of *P*_*e*_ on *pR* increases steeply in the presence of the LacI roadblock as the strength of *P*_*e*_ increases beyond its actual firing rate of 0.053 s^−1^ (termination rate held constant). Figure 7B shows a strong but less steep effect of reducing the termination rate on the TI of *P*_*e*_ on *pR* in the presence of the roadblock (*P*_*e*_ intrinsic firing rate constant). The weaker effect of termination rate compared to promoter firing rate is also apparent when both factors are varied (Fig. 7C). This is because occluding RNAPs are lost not only through termination but also by passage through the roadblock. Even in the absence of termination, the rate of loss of occluding RNAPs at the LacI roadblock can be as high as 0.026 s^−1^ (*k*_MD_).

## Discussion

The significance of these data and models are threefold; concerning how TI provides positive feedback in regulatory decision‐making, TI by dislodgement of transcriptional activators and mechanisms of promoter occlusion due to RNAP pausing.

### The role of TI in the lysis–lysogeny decision of bacteriophages λ and 186

Phages λ and 186 both utilize TI in their developmental decisions but do so in different ways. The main similarity is that the lytic promoter (λ *P*_*R*_ or 186 *pR*) is capable of substantially inhibiting the lysogeny‐establishing promoter (λ *P*_*RE*_ or 186 *pE*). However, the major mechanism of this TI and its magnitude are different in the two phages, with pause‐enhanced occlusion contributing to ~ 6‐fold TI in λ, and CII dislodgment contributing to at most fourfold TI in 186 (Fig. 5). The different mechanisms also result in a different response to CII. In λ, the inhibition by *P*_*R*_ is reasonably constant over a range of CII concentrations, while in 186, inhibition is maximal at low CII concentrations and decreases with increasing CII concentrations. Thus, in the presence of active *pR*, the level of expression of the lysogenic genes from 186 *pE* becomes highly sensitive to CII concentration, as increasing the CII concentration both directly activates *pE* and reduces its inhibition by *pR* (Fig. 4B). 186 *pR* also exerts TI on the convergent lysogenic promoter *pL* (Fig. 2). In this case TI is strong, ~ 6‐fold, and is primarily by the sitting duck mechanism 6, 18. In contrast, λ *P*_*R*_ and *P*_*R*M_ are back‐to‐back and cannot inhibit each other *in vivo* by any of the mechanisms shown in Fig. 1.

We note that our measurement of inhibition of *pE* by TI in 186 may be an underestimate because the decreased translation of the *pR* mRNA in our *pL* ^*−*^ constructs compared to the wild‐type case may have reduced the flux of RNAPs from *pR* at *pE*, due to the lack of ‘translation asymmetry’ in collisional TI. Our measurement of 2.3‐fold TI at maximal pE induction is similar to the 2.1‐fold value obtained for the same *pL* ^*−*^ mutant by 21. However, the TI value for the *pL+* case at maximal *pE* induction was 3.5‐fold in 21, when an estimate of the *pL* contribution to leftward transcription is subtracted. The interaction of the three promoters makes TI difficult to analyse but the result suggests that, in the presence of full *apl* translation, the TI of *pR* on *pE* at sub‐maximal CII activation may be higher than the ~ 4‐fold we observed. On the other hand, the presence of Apl would likely reduce this TI because of its repressive effect on *pR* (Fig. S3).

We note also that our previous modelling of TI by *P*_*R*_ on *P*_*RE*_ in λ did not include translation asymmetry due to the translation of *cro* and lack of translation of the *P*_*RE*_ RNA in the overlap region 7. Inclusion of translation asymmetry may allow the TI data to be explained with a weaker pause‐enhanced occlusion effect than we estimated.

Regardless of the mechanisms, the TI appears to provide for positive feedback by the lysogenic repressor CI, a feature that should sharpen the decision between lytic and lysogenic development. In λ, CI repression of *P*_*R*_ relieves TI on *P*_*RE*_, increasing *P*_*RE*_ transcription into the *cI* gene 7, 36. In 186, CI repression of *pR* relieves TI on *pL*, increasing lysogenic transcription 27, 28. In both cases, maximal relief of TI is possible because neither repressor presents a roadblock to RNAPs from the upstream promoter 27, 36. We expect that repression of *pR* by 186 CI will also relieve TI on *pE*. In addition, dislodgement of λ CI by the passing RNAPs does not impair repression of *P*_*R*_, implying fast binding kinetics for λ CI 36, while this has not been tested for 186 CI repression of *pR*. In λ, the relief of TI on *P*_*RE*_ by CI should provide the first CI positive feedback mechanism operating after infection, with repression of Cro and direct activation of *P*_*RM*_ being the second and third mechanisms 36. In 186, relief of TI is the only mechanism for positive feedback by CI at *pL* 27 and this appears to also be the case at *pE*. Thus, in these convergently evolved genetic switches, different mechanisms of TI are employed interchangeably but with a consistent function of establishing positive feedback.

### TI by dislodgement

In our previous study of relief of TI in λ, we found that two different repressors of *P*_*R*_, the natural CI repressor and a *Streptococcus pyogenes* dCas protein targeted to *P*_*R*_, behaved quite differently in response to transcription from *P*_*RE*_ 36. Both proteins strongly repressed *P*_*R*_, and neither protein acted as a roadblock to transcription from *P*_*RE*_ (dCas was targeted to the *P*_*RE*_ template strand), indicating that both were readily dislodged by elongating RNAPs from *P*_*RE*_. However, this dislodgement of dCas interfered with its repression of *P*_*R*_, but the dislodgement of λ CI did not 36. This difference was able to be explained by invoking a difference in binding kinetics, with slow binding kinetics by dCas and fast binding kinetics for λ CI.

The λ CII protein and the 186 CII protein provide a similar contrast in dislodgement sensitivity, but for transcription activators rather than transcription repressors. λ CII activation of *P*_*RE*_ was not affected by passing RNAPs from λ *P*_*R*_, while we found here that 186 CII activation of *pE* was inhibited by RNAPs from 186 *pR*. We showed that this could be explained if the binding kinetics of 186 CII were slow, such that its dislodgement by RNAPs significantly increased its rate of dissociation from DNA. Our results thus further emphasize the importance of DNA binding kinetics in the interaction between transcription factors and elongating RNAPs 2. Even though transcription factors and RNAP cannot be simultaneously bound to the same nucleotide, when binding kinetics are sufficiently rapid these entities can effectively ‘pass through’ one another with little evidence of interaction.

### Pausing‐enhanced occlusion

Our previous study of the interaction between λ *P*_*R*_ and *P*_*RE*_ provided evidence for pausing‐enhanced occlusion as a mechanism of TI 7. However, despite the similarities in promoter arrangement and regulatory requirements between the λ *P*_*R*_.*P*_*RE*_.*cII* system and the 186 *pR.pE.cII* system, the low TI exerted by 186 *pR* on *pE* at high CII levels indicates that 186 does not employ pause‐enhanced occlusion at *pE*. Instead, we were able to confirm the pause‐enhanced occlusion mechanism in a specifically designed synthetic (REO) construct by using a protein roadblock to pause RNAP, in contrast to the intrinsic pause mediated by λ *tR1*. In wild‐type cells, the LacI roadblock was responsible for a ~ 1.6‐fold enhancement of TI (1.96‐fold TI versus 1.24‐fold TI without the roadblock). As predicted, increasing the pausing time by using *mfd* ^*−*^ cells increased the enhancement of TI due to the roadblock, to ~ 3.4‐fold (5.02‐fold TI versus 1.49‐fold TI without the roadblock). Modelling indicated that the termination rate of paused RNAPs is a less critical factor in determining the magnitude of pause‐enhanced occlusion than the rate of firing of the promoter providing the occluding RNAPs. Thus, the weaker effect of the roadblock‐induced pause in the REO circuit compared to the *tR1*‐induced pause in the λ case is primarily due to the 3.3‐fold higher strength of λ *pR* compared to P2 *P*_*e*_. In general, strong pause‐enhanced occlusion, whatever the nature of the pause, requires a high rate of supply of RNAPs to the pause site to overcome their loss by termination and by passage through the roadblock. We expect the same principle to apply in other organisms.

## Author contributions

IBD and KES conceived and supervised the study; NH, MTC, IBD and KES designed experiments; NH and MTC performed experiments; NH, MTC and ACP did the modelling analysis; NH, MTC, IBD and KES analysed data; NH, IBD and KES wrote the manuscript; NH, ACP, IBD and KES made manuscript revisions.

## Funding

This work was supported by the Australian Research Council *via* a Discovery Early Career Researcher Award to NH (DE150100091) and Discovery Grants (DP150103009, DP160101450). NH was also supported in part by a Fellowship from Synthetic Biology Future Science Platform, Commonwealth Scientific and Industrial Research Organisation. ACP was supported by a James S. McDonnell Foundation Postdoctoral Fellowship.

## Supporting information


**Table S1.** Modelling parameters.
**Fig. S1.** Chromosomally integrated 186 TI and REO reporter constructs.
**Fig. S2.** The sequence of the phage 186 switch region.
**Fig. S3.** The *pL* ^*−*^ mutation reduces Apl translation.
**Fig. S4.** Sequence of the REO construct.
