# Supplementary material for: RNA polymerase pausing at a protein roadblock can enhance transcriptional interference by promoter occlusion
Source: FEBS Lett. 2019 Mar 29;593(9):903–17. doi: 10.1002/1873-3468.13365 (PMC6593788; doi:10.1002/1873-3468.13365)
Supplement: Supplementary file 1 — Table S1. Modelling parameters. Fig. S1. Chromosomally integrated 186 TI and REO reporter constructs. Fig. S2. The sequence of the phage 186 switch region. Fig. S3. The pL − mutation reduces Apl translation. Fig. S4. Sequence of the REO construct. [file FEB2-593-903-s001.pdf]

Supporting Information

RNA polymerase pausing at a protein roadblock can enhance transcriptional interference by promoter occlusion

Nan Hao, Michael Crooks, Adam C. Palmer, Ian B. Dodd and Keith E. Shearwin

Table S1: Modelling parameter

Parameter	Meaning	Values	References
**FIXED PARAMETERS**			
l	Length of elongating RNAP (bp)	30	[1]
v	Rate of RNAP elongation (bp s^{-1})	40	[1]
k_F^*	Intrinsic promoter firing rate (s^{-1}) [a]	186 *pR*: 0.0609 (LB), 0.0554 (MM)	This study
		186 *pE*: 0.1072 (LB) [b]	This study
		P2 P_e: 0.0527 (MM)	This study
k_{LacIon}	Binding rate of LacI to *lacOid* (s^{-1})	0.6474	[2]
$k_{LacIoff}$	Unbinding rate of LacI to *lacOid* (s^{-1})	0.0004	[2]
**FITTED PARAMETERS**			
k_{CIIoff}	Unbinding rate of CII to its binding site at *pE* (s^{-1})	0.025	This study
K_{CIIon}	Binding rates of CII to its binding site at *pE* (s^{-1}) [0, 20, 35, 50, 65, 80, 100, 130, 160, 200, 250, 300] µM IPTG	0.0000, 0.0006, 0.0023, 0.0068, 0.0170, 0.0341, 0. 0501, 0.1126, 0.1503, 0.2843, 0.3242, 3.6621	This study
α	Aspect ratio	186 *pR*: 0.0001	This study
		186 *pE*: 1.0	This study
		P2 P_e: 1.0	This study
k_O	Rate of open complex formation (s^{-1})	186 *pR*: 0.0609 (LB), 0.0554 (MM)	This study
		186 *pE*: 0.2143 (LB)	This study
		P2 P_e: 0.1054 (MM)	This study
k_E	Rate of transition from an open to an elongating complex (s^{-1})	186 *pR*: 609.0 (LB), 554.0 (MM)	This study
		186 *pE*: 0.2143 (LB)	This study
		P2 P_e: 0.1054 (MM)	This study
k_T	Rate of RNAP termination (s^{-1})	*wt*: 0.0659	[2]
		mfd KO: 0.02	This study
k_{SD}	Rate of dislodgement by a single RNAP (s^{-1})	*wt*: 0.0015	[2]
		mfd KO: 0.0033	[2]
k_{MD}	Rate of dislodgement by multiple RNAPs (s^{-1})	*wt*: 0.0259	[2]
		mfd KO: 0.011	[2]
K_{dT}	Rate of delayed termination following RNAPs collision (s^{-1})	*wt*: instantaneous	[2]
		mfd KO: 0.02	This study

[a] The intrinsic promoter firing rate was calculated using Equation 1, and was adjusted to account for self-occlusion (Materials and Methods)
[b] This is the intrinsic firing rate of 186 *pE* when fully activated by CII, basal *pE* has negligible activity

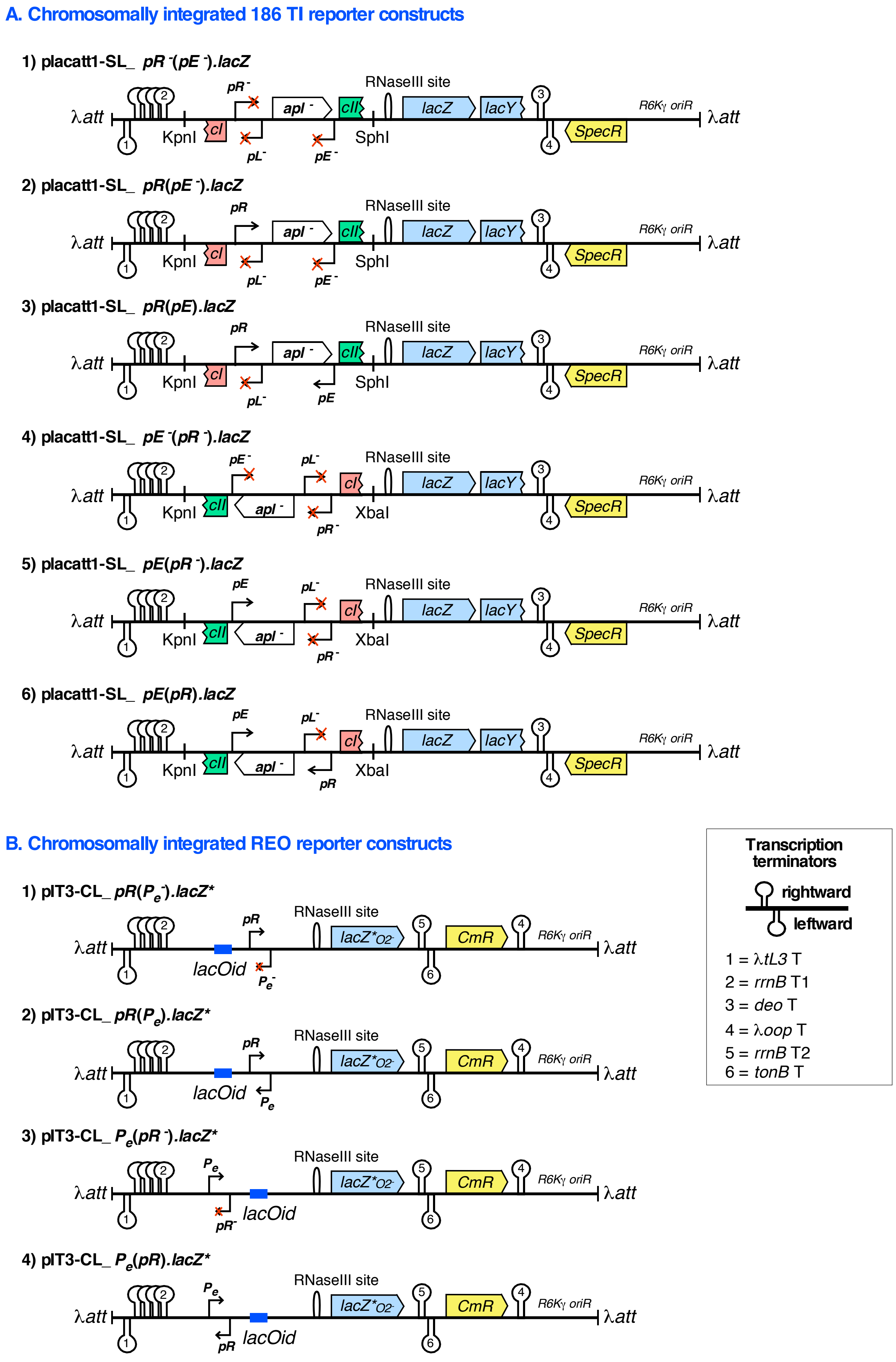

Fig. S1. Chromosomally integrated 186 TI and REO reporter constructs.

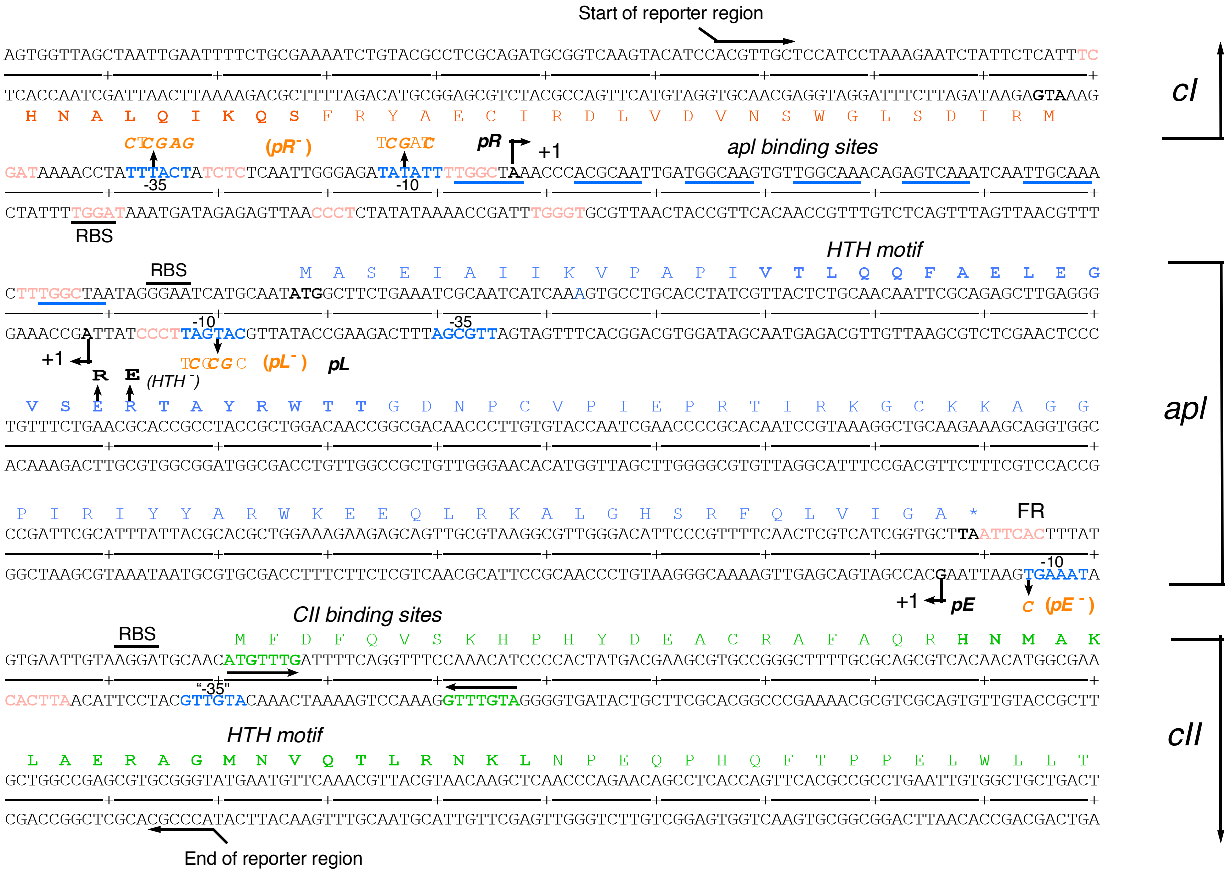

Fig. S2. The sequence of the phage 186 switch region. The -10 and -35 sites of the *pR*, *pE*, and *pL* promoters are blue and in bold. Promoter inactivating mutations are in red and shown above the wild-type sequence. The direction and start site (+1) of transcription are marked with an arrow. The ribosome binding sites (RBS) sequences are underlined, and the translated sequence of coding regions is shown above the DNA sequence, with helix-turn-helix (HTH) motifs in bold. CII binding site inverted repeats are shown in green, CI binding sites are shown in red, and the Apl binding sites are denoted with blue underlines.

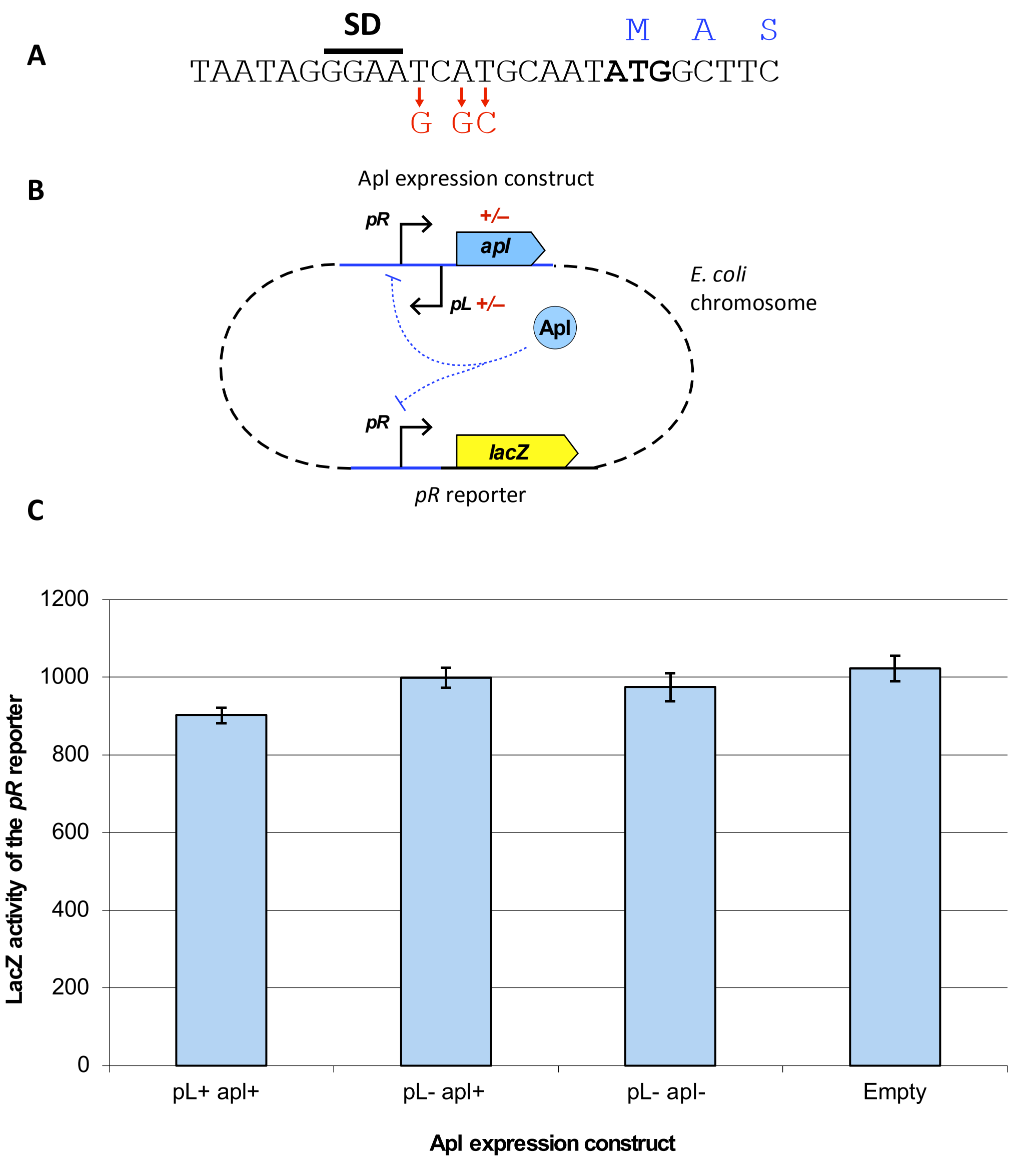

Fig. S3 The *pL*$^{-}$ mutation reduces Apl translation.
A. The base changes to the Apl ribosome binding site (see Fig. S2) lie between the Shine-Delgarno sequence and the *apl* ATG. Using the Salis lab RBS calculator [3,4], the mutated *apl* RBS at ~549au, is ~6-fold weaker than the wild-type *apl* RBS.
B. Single-copy chromosomally integrated expression and reporter constructs containing wild-type or mutant *pL* and wild-type or mutant *apl*. Apl produced from the wild-type construct represses *pR*. All constructs contain all 7 Apl binding sites at *pR.pL.*
C. Mean and 95% confidence limits for the *pR* reporter LacZ units in the presence of the four expression constructs (*n*=20, 24, 16 and 24, respectively). There was no significant difference ($p >$ 0.05) for intercomparisons of *pL-apl+*, *pL-apl-* and empty, and $p < 0.05$ for comparisons of *pL+apl+* with any of the others (one-tailed *t*-tests for unpaired samples of unequal means).

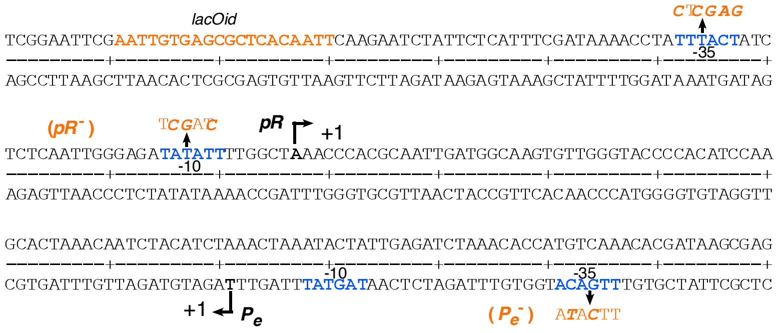

Fig. S4. Sequence of the REO construct. Promoter mutations are indicated in red above the sequence.

SUPPLEMENTARY REFERENCES:

[1] Sneppen, K., Dodd, I.B., Shearwin, K.E., Palmer, A.C., Schubert, R.A., Callen, B.P. and Egan, J.B. (2005). A mathematical model for transcriptional interference by RNA polymerase traffic in Escherichia coli. J Mol Biol 346, 399 -409.

[2] Hao, N., Krishna, S., Ahlgren -Berg, A., Cutts, E.E., Shearwin, K.E. and Dodd, I.B. (2014). Road rules for traffic on DNA -systematic analysis of transcriptional roadblocking in vivo. Nucleic Acids Res 42, 8861 -72.

[3] Salis, H.M., Mirsky, E.A. and Voigt, C.A. (2009). Automated design of synthetic ribosome binding sites to control protein expression. Nat Biotechnol 27, 946 -50.

[4] Espah Borujeni, A., Channarasappa, A.S. and Salis, H.M. (2014). Translation rate is controlled by coupled trade -offs between site accessibility, selective RNA unfolding and sliding at upstream standby sites. Nucleic Acids Res 42, 2646 -59.
